# Assessing lactate stability at the minimum lactate steady state velocity in male trained middle-distance runners

**DOI:** 10.1371/journal.pone.0344573

**Published:** 2026-03-06

**Authors:** Seyed Houtan Shahidi

**Affiliations:** Faculty of Sports Sciences, Department of Sports Coaching, Istanbul Gedik University, Istanbul, Turkey; South China University of Technology, CHINA

## Abstract

**Objectives:**

This study investigated the physiological behavior of the running velocity associated with the Minimum Lactate Steady State (vMLaSS), derived from a 6 × 800-m interval protocol, and examined whether this intensity produced stable metabolic and lactate responses during a 30-minute constant-load validation run in trained endurance runners.

**Methods:**

Fifteen trained male middle- and long-distance runners completed a graded treadmill test to determine maximal oxygen uptake. Following a supramaximal sprint to induce hyperlactatemia, each athlete performed a 30-minute constant-load run at a velocity derived from the lactate-minimum approach. Following a supramaximal sprint to induce hyperlactatemia, each athlete performed a 30-minute constant-speed run at their individually determined MLaSS velocity. Blood lactate samples were collected at 10-minute intervals, and breath-by-breath cardiopulmonary variables were continuously recorded. Lactate kinetics were analyzed using a Friedman test with Wilcoxon signed-rank post-hoc comparisons (*p* < 0.05).

**Results:**

Blood lactate exhibited significant time-dependent fluctuations during the 30-minute trial (Friedman χ² (3) = 28.72, *p* < 0.001). Lactate increased sharply by minute 10, declined at minute 20, and rose again at minute 30, exceeding the classical MLSS criterion of ≤1 mmol·L ⁻ ¹ change during the final 20 minutes. In contrast, cardiopulmonary variables remained stable throughout V̇O₂ (3.43 ± 0.11 L·min ⁻ ¹; *p* = 0.86) and V̇CO₂ (3.21 ± 0.14 L·min ⁻ ¹; *p* = 0.91). Carbohydrate oxidation predominated (214.5 ± 19.3 g·h ⁻ ¹), whereas fat oxidation remained minimal (–0.9 ± 2.7 g·h ⁻ ¹).

**Conclusion:**

Despite stable cardiorespiratory and substrate-utilization profiles, the significant variability in blood lactate concentration during the 30-minute constant-load run indicates that the running velocity derived from the lactate-minimum approach did not elicit a lactate steady state in this trained cohort. These findings suggest that physiological responses at the MLaSS-derived intensity may differ from classical steady-state expectations in highly trained endurance runners and highlight the need for direct MLSS verification in future studies.

## 1. Introduction

Endurance performance is governed by the balance between aerobic capacity and metabolic regulation across varying intensities. Seminal work by Wasserman et al. introduced the concepts of anaerobic threshold (AT) and respiratory compensation point (RCP), which reflect transitions between aerobic and anaerobic metabolism and mark key demarcations of exercise intensity [1]. These thresholds align closely with lactate dynamics, offering insight into substrate utilization, acid-base regulation, and sustainable intensity domains in endurance athletes [2,3]. Therefore, identifying reliable markers of sustainable effort, such as the lactate threshold, minimum lactate steady state (MLaSS), or maximal lactate steady state (MLSS), remains a cornerstone of endurance training prescription and performance assessment.

The MLaSS is derived from the Lactate Minimum Test (LMT), originally proposed by Tegtbur et al. [4]. The LMT does not assess a physiological steady state but identifies the minimum point of a U-shaped blood lactate curve following induced hyperlactatemia via a supramaximal sprint [5,6]. This lactate minimum has been proposed as an indirect estimate of the exercise intensity associated with MLSS, although it does not confirm a verified balance between lactate production and clearance [7]. Although previous investigations have attempted to evaluate the validity of the lactate minimum approach using blood lactate responses alone, such methods provide only a partial view of the underlying physiology and therefore limit the accuracy with which sustainable exercise intensity can be interpreted [8,9]. In the present study, we incorporated continuous cardiopulmonary assessment to obtain real-time measurements of oxygen uptake (V̇O₂), carbon dioxide output (V̇CO₂), and substrate oxidation rates during both laboratory and field running. This integrated approach enhances the interpretability of the lactate data by allowing the concurrent evaluation of metabolic behavior, particularly the shift from mixed or fat-supported metabolism toward predominantly glycolytic energy production, a transition closely associated with increased lactate appearance [10]. Because lactate kinetics are inseparable from the interaction of aerobic and anaerobic energy systems, combining metabolic and lactate measurements provides a more physiologically robust evaluation of whether the intensity identified by the lactate minimum truly reflects a sustainable metabolic steady state [1,3,11,12].

To our knowledge, no previous investigation has evaluated the validity of MLaSS by simultaneously examining metabolic responses and lactate kinetics in well-trained endurance runners. Accordingly, the present study aimed to determine whether the running velocity identified at the lactate-minimum point elicits stable lactate and cardiorespiratory responses during prolonged constant-load exercise in well-trained endurance runners.

## 2. Materials and methods

This study was approved by the Istanbul Gedik University Ethics Committee and conducted in accordance with the ethical standards outlined in the Declaration of Helsinki. The ethics approval number is E-56365223-050.04-2025.137548.76. Participant recruitment was conducted from 01/04/2025–01/06/2025. Before participation, all individuals were provided with detailed written and verbal information about the study’s purpose, procedures, potential risks, and their rights, including the right to withdraw at any point without consequence. Written informed consent was obtained from all participants before data collection began. No form of coercion was used, and participation was entirely voluntary. No personally identifying information has been included in the manuscript, and all data were anonymized for analysis and reporting.

### 2.1. Participants

Fifteen well-trained adult male middle- and long-distance runners specializing in the 1500-m and 3000-m events volunteered to participate in this study. All participants were competitive athletes with regular training backgrounds and completed all testing procedures.

Their average personal best performances were 4:05 ± 0:11 min for the 1500-m and 8:54 ± 0:17 min for the 3000-m. All participants trained 60–90 km per week and completed structured training programs that included interval training, tempo runs, aerobic base sessions, strength and conditioning (1–2 sessions per week), and injury-prevention exercises. Inclusion criteria required a minimum of three years of competitive experience, stable training in the month preceding testing, and absence of injury or illness. Exclusion criteria included musculoskeletal injury within the previous six months, use of supplements known to affect lactate kinetics, and any known cardiovascular, metabolic, or respiratory disorders. To standardize physiological conditions, participants were instructed to avoid strenuous exercise for 48 hours, refrain from caffeine for 12 hours, and alcohol for 24 hours. Only male athletes were included to control for sex-related physiological variability. An a priori power analysis (G*Power 3.1) indicated that 12 participants were sufficient for the repeated-measures design; therefore, 15 athletes were recruited to allow for potential dropouts [13].

### 2.2. Familiarization

All participants completed a dedicated familiarization session one week prior to data collection to ensure full understanding of the testing procedures and to minimize learning effects. During this session, athletes practiced treadmill running, lactate sampling, and breathing through the mask of the portable metabolic measurement system (MetaMax 3B, Cortex Biophysik GmbH, Leipzig, Germany; software version 1.6). All tests were performed in the pre-competitive season under controlled laboratory environmental conditions (temperature: 20–22°C; humidity: 45–55%). Participants were instructed to arrive in their usual training clothes and running shoes and maintain normal hydration. No participant used medications known to influence metabolism or cardiorespiratory function. A standardized 10-minute warm-up (light jogging and dynamic drills) was performed before each test. All procedures were supervised by certified exercise physiologists with extensive experience in cardiopulmonary and lactate-based testing, ensuring adherence to ACSM laboratory guidelines and high procedural reliability.

### 2.3. Anthropometric assessment

All baseline assessments were conducted in the morning hours within the Sports Performance Laboratory, following a minimum 8-hour fasting period in accordance with the guidelines established by the International Society for the Advancement of Kinanthropometry [14]. Height (cm) and body mass (kg) were measured using a stadiometer and digital scale (Seca 284, Seca GmbH & Co. KG, Hamburg, Germany). Body composition, including body fat percentage, was assessed via multifrequency bioelectrical impedance analysis using the Tanita MC-980 Body Composition Analyzer (Tanita Corporation, Tokyo, Japan). All measurements were taken by the same trained investigator to minimize inter-rater variability.

### 2.4. Maximal oxygen uptake (VO₂max)

Following anthropometric assessments, participants completed a graded ramp test to determine maximal oxygen uptake (V̇O₂max). The test was conducted on a high-performance, motorized treadmill (Trackmaster® TMX425C, Full Vision Inc., Newton, Kansas, USA), known for its precise speed calibration and stability, suitable for high-intensity running assessments. The protocol began with a standardized 10-minute warm-up at 8 km/h, followed by a ramp protocol starting at 10 km/h with 1 km/h increments every minute until volitional exhaustion, in accordance with the protocol described by [15]. The V̇O₂, V̇CO₂, respiratory exchange ratio (RER), and minute ventilation (V̇E) were continuously recorded using a portable metabolic analyzer (MetaMax® 3B, Cortex Biophysik GmbH, Leipzig, Germany), which was calibrated for both volume and gas concentration before each test using a 3-liter syringe and certified reference gas (15% O₂, 5% CO₂, balance N₂). Heart rate was monitored using a high-accuracy telemetry sensor (Polar H10, Polar Electro Oy, Kempele, Finland). VO₂max was determined as the highest 30-second rolling average of V̇O₂. The criteria for VO₂max attainment included: A plateau in oxygen uptake despite increasing workload, an RER ≥ 1.10, and Volitional exhaustion with inability to continue. Fat (FATox) and carbohydrate (CHOox) oxidation rates were computed using the non-protein stoichiometric equations, under the assumption of negligible urinary nitrogen excretion: FATox (g·min ⁻ ¹) = 1.695 × V̇O₂ – 1.701 × V̇CO₂; CHOox (g·min ⁻ ¹) = 4.344 × V̇CO₂ – 3.061 × V̇O₂ [16].

### 2.5. Determination of 3000-m vV̇O₂max

The 3000-m running test was used to estimate the running velocity associated with VO₂max (vV̇O₂max), following widely used field protocols in endurance performance research [6]. Forty-eight hours after the laboratory VO₂max test, each athlete performed an individual 3000-m time trial on a standardized 400-m outdoor track under controlled environmental conditions (temperature 18–22°C, humidity 50–60%, wind <5 km·h ⁻ ¹). Participants were instructed to run the distance as fast as possible, maintaining an even pace without sprinting at the start or finish. Pacing was supported using GPS-enabled running watches (Garmin Forerunner 945, USA), and athletes received standardized verbal encouragement. The mean running velocity across 3000 m was calculated as vV̇O₂max = distance (3000 m) / time (s).

### 2.6. Minimum lactate steady state (MLaSS) protocol

The MLaSS protocol followed procedures adapted from Simoes et al., combining an initial sprint-induced hyperlactatemia with a series of submaximal running bouts [6,7]. The protocol began with a 500-m all-out sprint to elevate lactate concentrations. After an 8-minute passive recovery, participants completed six consecutive 800-m bouts at intensities corresponding to 86%, 88%, 90%, 92%, 94%, and 97% of each athlete’s vV̇O₂max (The vV̇O₂max was calculated directly from the 3000-meter running performance). The selected range (86–97%) was intended to span the upper heavy domain and extend into the severe domain. Pace control was achieved using auditory cues every 100 m (pre-programmed metronome beeps) and GPS-based pacing feedback (Garmin Forerunner 945, Garmin Ltd., Olathe, Kansas, USA). Blood samples were collected from the earlobe during the seventh minute of recovery following the sprint and during the final minute of each 800-m stage. Lactate values were plotted against running velocity, and a second-order polynomial function was fitted to identify the lactate minimum point, which was defined as the MLaSS velocity.

### 2.7. 30-minute validation run at minimum lactate steady state (MLaSS) velocity

A continuous 30-minute constant-load run was performed to examine the physiological responses to the running velocity identified using the lactate-minimum approach during the 6 × 800-m protocol. Blood lactate responses were evaluated using classical MLSS criteria as a reference framework to describe lactate stability during prolonged exercise, without implying direct confirmation of MLSS [6,7]. All athletes completed the test on a 400-m synthetic outdoor track under standardized environmental conditions (temperature 18–22°C; humidity 50–60%). The target speed corresponded to each athlete’s individually determined MLaSS velocity (vMLaSS) from the incremental 6 × 800-m test. Running pace was tightly controlled using auditory cues every 100 m (pre-programmed metronome beeps) and GPS feedback (Garmin Forerunner 945, Garmin Ltd., USA), ensuring deviations stayed within ± 2% of the prescribed speed. To maintain validity relative to MLSS standards, blood lactate samples were taken from the earlobe at the 10th, 20th, and 30th minutes of the run (Lactate Scout 4, EKF Diagnostics, Germany). A lactate change ≤1.0 mmol·L ⁻ ¹ between 10 and 30 minutes was considered indicative of a steady state, consistent with classical MLSS criteria. Participants were required to complete the full 30 minutes.

### 2.8. Statistical analysis

Descriptive statistics are presented as mean ± standard deviation for all physiological variables, including V̇O₂, V̇CO₂, RER, blood lactate concentration, and substrate oxidation rates. Normality was assessed using the Shapiro-Wilk test. Because lactate data violated normality assumptions, nonparametric analyses were applied. Time-dependent lactate responses during the 30-minute MLaSS run were evaluated using the Friedman test, with Wilcoxon signed-rank tests used for post-hoc comparisons where appropriate. Lactate stability was assessed using the classical MLSS criterion of a < 1.0 mmol·L ⁻ ¹ increase between minutes 10 and 30. Effect sizes were calculated as r = Z/√n, where r = .10 (small),.30 (moderate), and .50 (large) [17]. All analyses were conducted using OriginPro 2025, with statistical significance set at *p* < 0.05.

## 3. Results

### 3.1. Anthropometric and oxygen uptake

Fifteen trained male middle- and long-distance runners (age: 21.3 ± 2.4 years, body mass: 67.8 ± 5.9 kg, height: 176.2 ± 6.8 cm, body fat: 9.4 ± 2.1%) completed an incremental ramp test to determine maximal oxygen uptake. The mean relative V̇O₂max was 66.4 ± 2.8 mL·kg ⁻ ¹·min ⁻ ¹.

### 3.2. 3000-m time trial and metabolic response

**Fig 1:**
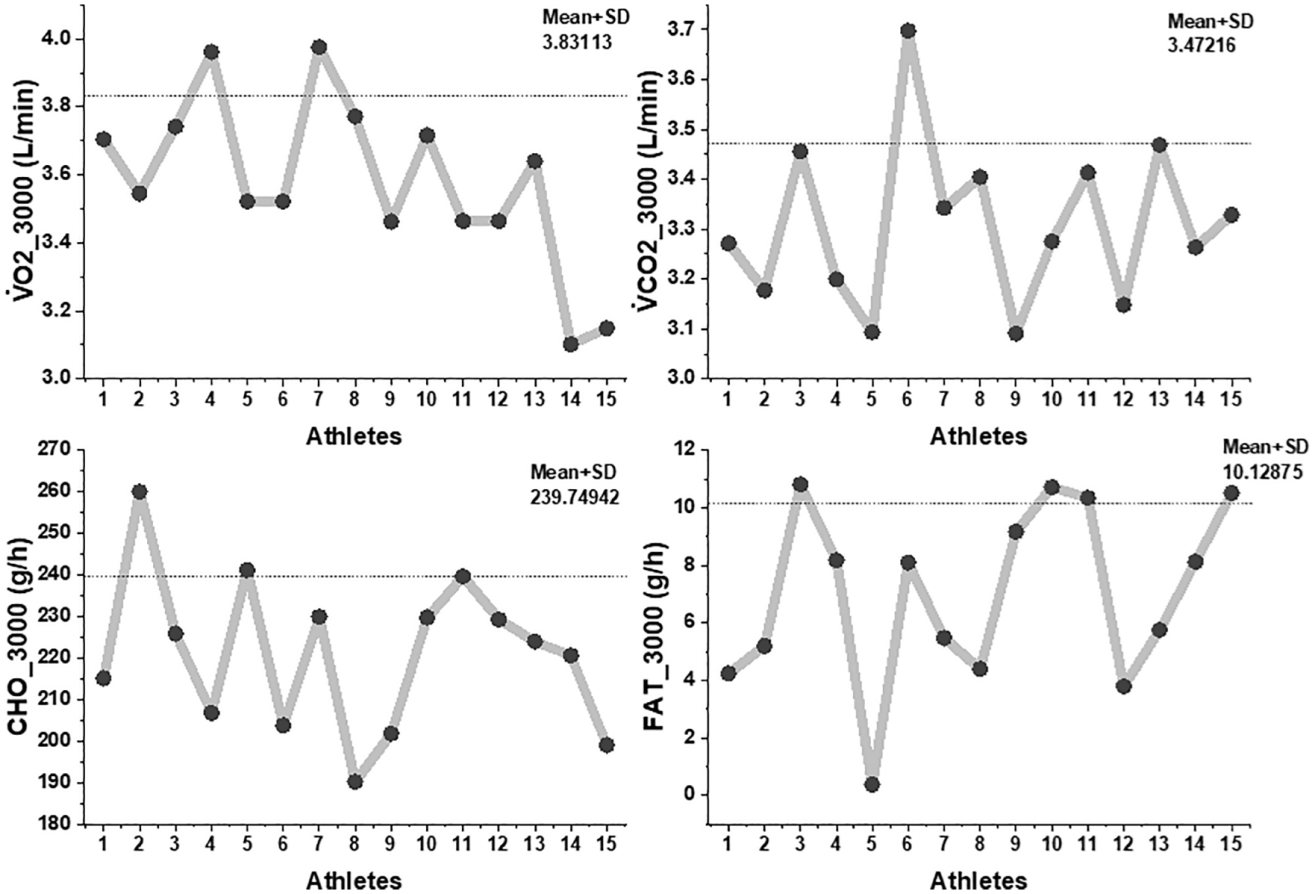
Interindividual variability in oxygen consumption, carbon dioxide production, and substrate oxidation during 3000-m time trial. Note. This multi-panel figure illustrates individual responses (n = 15) in four key physiological parameters measured during a 3000-meter time trial: Top left (V̇O₂_3000): Oxygen uptake (mean ± SD = 3.83 ± 0.25 L·min ⁻ ¹); Top right (V̇CO₂_3000): Carbon dioxide output (3.47 ± 0.23 L·min ⁻ ¹); Bottom left (CHO_3000): Carbohydrate oxidation rate (239.75 ± 21.3 g·h ⁻ ¹); Bottom right (FAT_3000): Fat oxidation rate (10.13 ± 1.8 g·h ⁻ ¹). Dashed horizontal lines represent the group mean.

The athletes completed the 3000-m time trial in 9:14 ± 0:22 min, corresponding to an average running velocity of 3.24 ± 0.08 m/s (3:05 ± 0:07 min·km ⁻ ¹). This velocity represented 85–90% of vV̇O₂max. Gas-exchange data, collected for physiological characterization, showed that mean V̇O₂ during the trial was 3.58 ± 0.25 L/min, with V̇CO₂ of 3.39 ± 0.21 L/min. Substrate-oxidation estimates indicated a clear predominance of carbohydrate metabolism (CHOox: 226.1 ± 18.3 g/h), while fat oxidation remained minimal (6.9 ± 3.7 g/h) as shown in Fig 1.

### 3.3. Minimum lactate steady state (MLaSS) assessment

**Fig 2:**
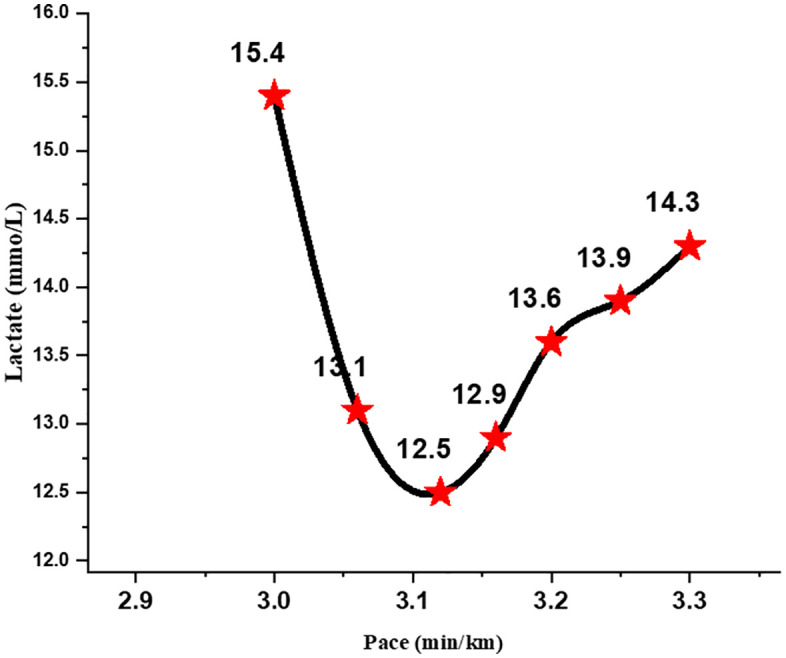
Lactate-pace relationship during the 6 × 800-m minimum lactate protocol.

The maximal 500-m sprint resulted in a peak blood lactate concentration of 14.3 ± 0.6 mmol·L ⁻ ¹, which declined slightly to 13.9 ± 0.5 mmol·L ⁻ ¹ after the standardized 8-minute passive recovery. During the subsequent 6 × 800-m incremental protocol, lactate demonstrated a clear U-shaped pattern. Lactate values decreased across the initial stages and reached a nadir of 12.5 ± 0.4 mmol·L ⁻ ¹ at a running velocity of 3:11 ± 0:08 min·km ⁻ ¹ (Fig 2).

**Fig 3:**
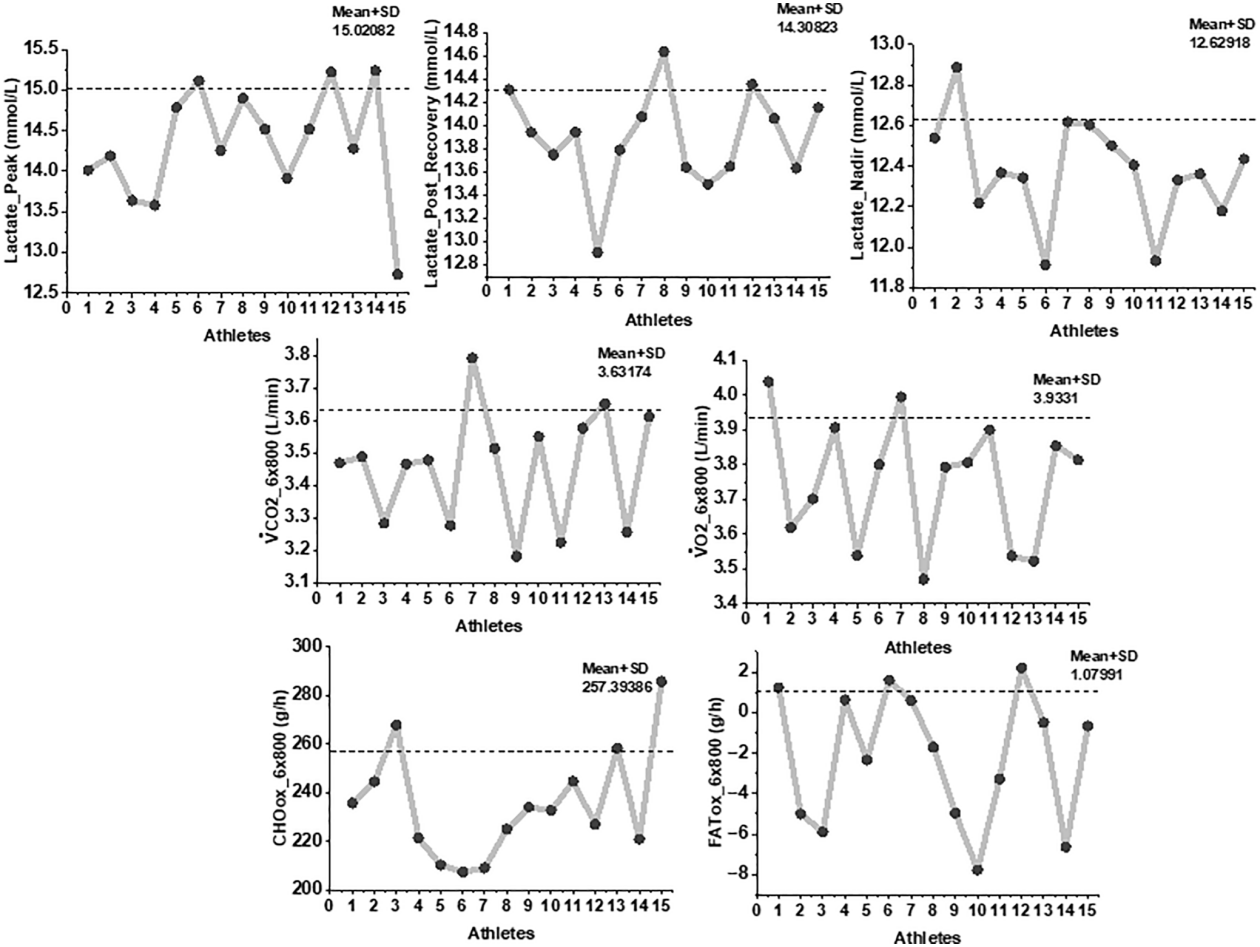
Individual variability in lactate kinetics, MLaSS running velocity, glycolytic capacity, and metabolic responses. Note. Individual athlete values for key physiological variables measured during the Minimum Lactate Steady State (MLaSS) protocol. Panels include: Peak blood lactate, Post-recovery lactate, Lactate nadir, Running velocity at MLaSS (vMLaSS), Maximal lactate production rate (VLamax), oxygen uptake (V̇O₂), carbon dioxide output (V̇CO₂), Carbohydrate oxidation (CHO), and Fat oxidation (FAT). Each point represents one of the 15 athletes, with dashed lines indicating group mean ± SD.

The Friedman test indicated a significant overall effect of running stage on lactate concentration (χ² (3) = 26.54, *p* < 0.001). Post-hoc Wilcoxon tests confirmed a significant reduction from post-recovery to the nadir stage (Z = –3.41, *p* < 0.01, r = 0.88), followed by a significant increase at higher velocities (Z = –3.29, *p* < 0.01, r = 0.85). The velocity corresponding to the individual MLaSS point averaged 3:07 ± 0:03 min·km ⁻ ¹ across participants. Peak lactate following the final (sixth) 800-m repetition reached 15.4 ± 0.5 mmol·L ⁻ ¹, representing a statistically significant increase from the nadir (Z = –3.46, *p* < 0.01, r = 0.89). Gas-exchange measurements during the incremental bouts showed peak values of 3.76 ± 0.18 L·min ⁻ ¹ for V̇O₂ and 3.42 ± 0.20 L·min ⁻ ¹ for V̇CO₂. Carbohydrate oxidation predominated throughout the protocol (226.8 ± 21.7 g·h ⁻ ¹), whereas fat oxidation remained minimal (–1.4 ± 4.2 g·h ⁻ ¹) as shown in Fig 3. A small rise in fat oxidation during the fifth interval was observed, but did not reach statistical significance (χ² (5) = 5.21, *p* = 0.39).

### 3.4. 30-minute validation run at minimum lactate steady state (MLaSS) velocity

**Fig 4:**
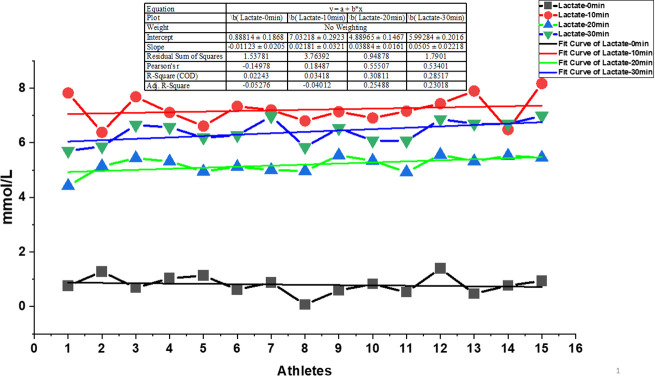
Linear trends in blood lactate concentrations across 0, 10, 20, and 30 minutes during the minimum lactate steady state (MLaSS) stage. Note. Blood lactate concentrations were measured at 0, 10, 20, and 30 minutes during the Minimum Lactate Steady State (MLaSS) running stage for 15 trained athletes. Each color-coded line represents a different sampling time (0 min: black, 10 min: red, 20 min: blue, 30 min: green). Individual athlete values are shown alongside their respective linear regression fit curves, with slope, intercept, R^2^, adjusted R^2^, Pearson’s r, and residual sum of squares presented in the inset table.

At the individualized MLaSS velocity (90.1 ± 1.8% vV̇O₂max, equivalent to 3:07 ± 0:03 min·km ⁻ ¹), the 30-minute continuous validation run produced a distinct time-dependent lactate profile (Fig 4).

**Fig 5:**
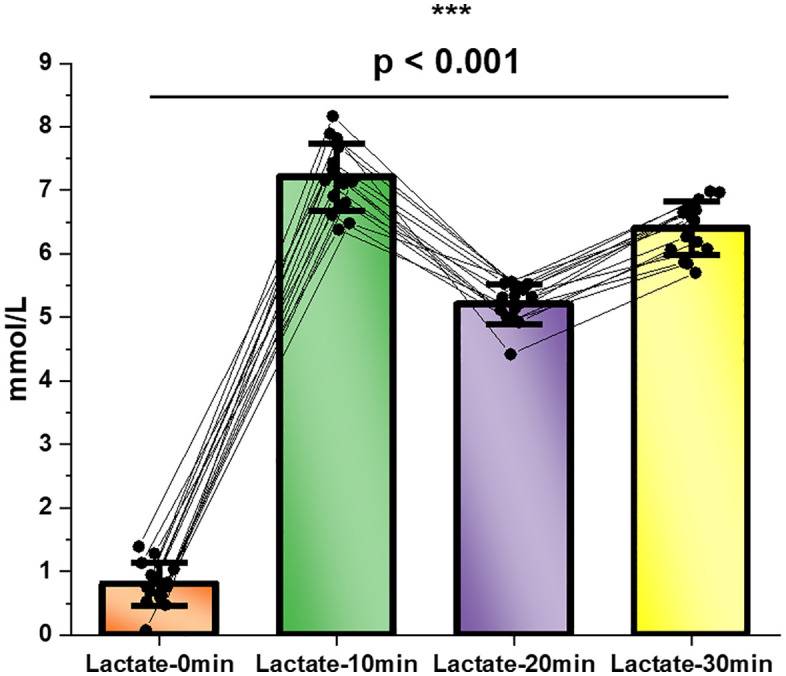
Lactate responses across 0, 10, 20, and 30 minutes during the minimum lactate steady state (MLaSS) stage. Note. Individual lactate responses were measured at four time points (0, 10, 20, and 30 minutes) during the Minimum Lactate Steady State (MLaSS) running stage. Bars represent mean ± SD, while connecting lines show individual athlete trajectories (n = 15). Lactate increased sharply from baseline to 10 minutes (p < 0.001), followed by a reduction toward 20 minutes and a slight rise again at 30 minutes.

A Friedman test indicated a significant main effect of time on lactate concentration (χ² (3) = 28.72, *p* < 0.001). Lactate rose steeply from resting baseline (0.9 ± 0.3 mmol·L ⁻ ¹) to minute 10 (7.1 ± 0.5 mmol·L ⁻ ¹). Post-hoc Wilcoxon signed-rank tests confirmed that this increase was significant (Z = –3.52, *p* < 0.001, r = 0.91). Between minutes 10 and 20, lactate declined to 5.2 ± 0.4 mmol·L ⁻ ¹, representing a statistically significant reduction (Z = −3.41, *p* < 0.001, r = 0.88) as shown in Fig 5.

By minute 30, lactate increased again to 6.2 ± 0.6 mmol·L ⁻ ¹, with significant differences relative to both minute 20 (Z = −3.29, *p* = 0.001, r = 0.85) and minute 10 (Z = −3.08, *p* = 0.002, r = 0.80). Despite these fluctuations, the absolute change between minutes 10 and 30 remained within the established MLaSS criterion (<1 mmol·L ⁻ ¹). At the individual level, all athletes demonstrated a lactate change ≤1.0 mmol·L ⁻ ¹ over this interval, with no evidence of progressive upward drift (Fig 4).

**Fig 6:**
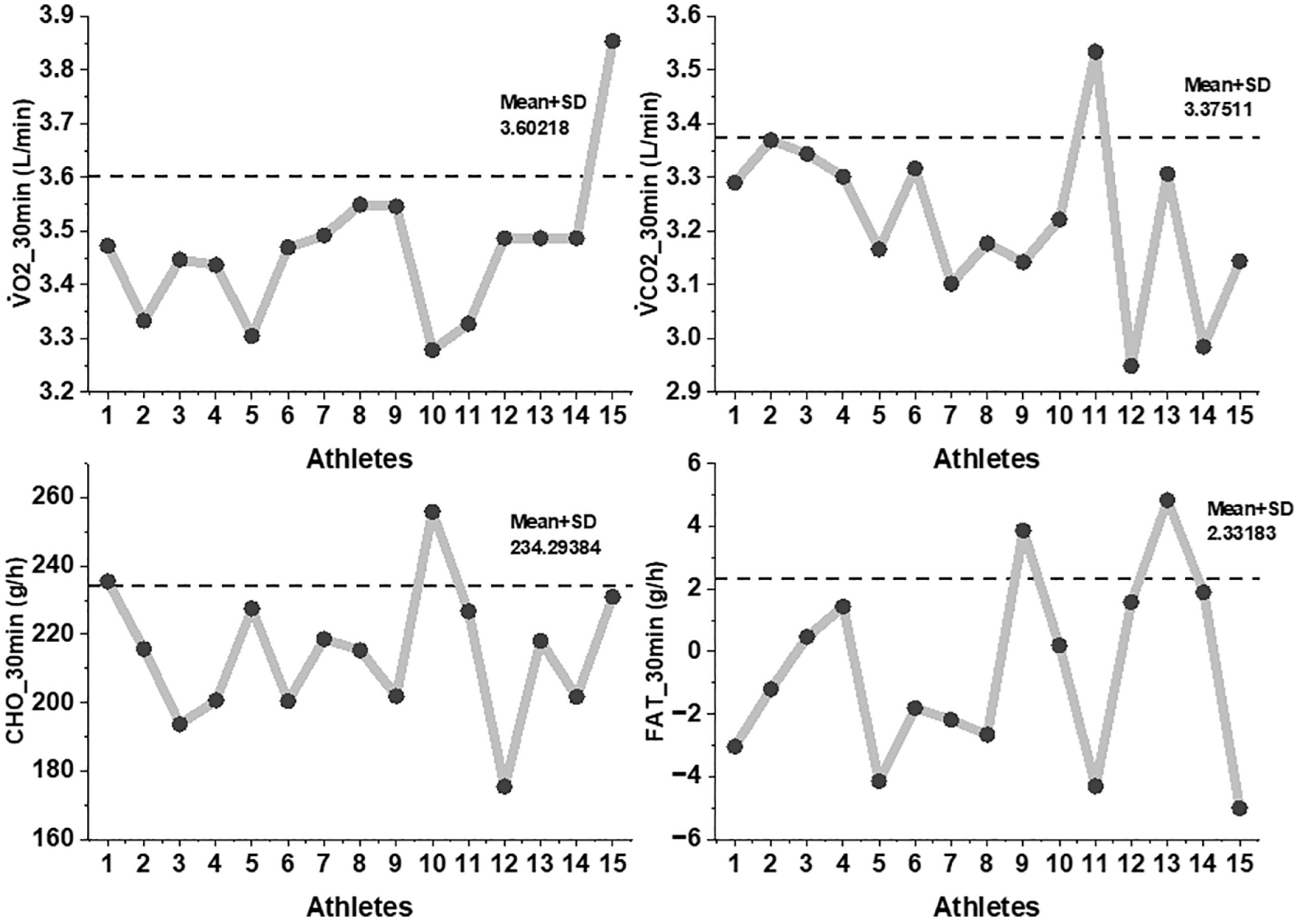
Individual V̇O_2_, V̇CO_2_, carbohydrate, and fat oxidation responses during the final 30 minutes of the minimum lactate steady state (MLaSS). Note. Individual athlete data for oxygen uptake (V̇O₂), carbon dioxide output (V̇CO₂), carbohydrate oxidation (CHO), and fat oxidation (FAT) measured during the 30 minutes of the Minimum Lactate Steady State (MLaSS) running stage. Each point represents one of 15 trained athletes, with dashed horizontal lines indicating group mean ± SD.

Oxygen uptake (V̇O₂) remained constant at 3.43 ± 0.11 L·min ⁻ ¹, with no significant time effect (Friedman χ² (2) = 1.84, p = 0.40), and carbon dioxide output (V̇CO₂) remained similarly steady at 3.21 ± 0.14 L·min ⁻ ¹ (χ² (2) = 2.12, *p* = 0.35). Substrate utilization was dominated by carbohydrate metabolism, with mean CHO oxidation of 214.5 ± 19.3 g·h ⁻ ¹, while fat oxidation remained negligible (−0.9 ± 2.7 g·h ⁻ ¹). No significant changes in CHO or FAT oxidation were detected across time (*p* > 0.05 for all pairwise tests, Fig 6).

## 4. Discussion

The primary aim of this study was to characterize lactate and physiological responses during a 30-minute constant-load run performed at a running velocity derived from the minimum lactate approach in trained middle-distance runners. The principal finding is that this MLaSS-derived intensity did not elicit a stable blood lactate profile across the duration of exercise. Although the running velocity corresponded to approximately 90% of vV̇O₂max and cardiopulmonary variables remained stable, blood lactate concentration exhibited significant time-dependent fluctuations and exceeded the classical MLSS criterion of a ≤ 1 mmol·L ⁻ ¹ increase during the final 20 minutes (Figs 3 and 4). The Friedman test confirmed significant time-dependent fluctuations in lactate concentration (χ² (3) = 28.72, p < 0.001), and Wilcoxon post-hoc analyses demonstrated a rise at minute 10, a mid-test decline at minute 20, and a renewed increase by minute 30 (Fig 4).

Previous studies have suggested that the lactate-minimum concept approximates MLSS in moderately trained athletes [5–7,8,9]. However, our findings do not align with these assumptions when applied to highly trained endurance runners. The runners exhibited high post-sprint lactate peaks (14.3 ± 0.6 mmol·L ⁻ ¹; Fig 2). These characteristics may indicate a cohort with well-developed anaerobic capabilities concurrent with high aerobic fitness, typical of middle-distance runners who rely heavily on both oxidative and glycolytic pathways [18,19]. Consequently, the “nadir” point during MLaSS testing may falsely appear steady because lactate clearance temporarily exceeds production early in the protocol. This may explain why the nadir in our MLaSS test (12.5 ± 0.4 mmol·L ⁻ ¹) occurred during the interval stages but could not be maintained during the continuous 30-min validation. The metabolic “false steady state” created by the preceding high-intensity sprint may support critiques that lactate-minimum methods are sensitive to prior exercise, recovery duration, and residual lactate kinetics [4,20,21].

Despite clear instability in blood lactate concentrations, both V̇O₂ (3.43 ± 0.11 L/min) and V̇CO₂ (3.21 ± 0.14 L/min) remained relatively stable during the 30-minute validation run. However, this stability in cardiorespiratory variables should not be interpreted as evidence of a metabolic steady state. Elite endurance runners are characterized by rapid pulmonary oxygen uptake kinetics, high mitochondrial enzyme activity, and enhanced peripheral oxygen extraction, enabling oxygen delivery to closely match metabolic demand even as glycolytic flux increases [22–24]. The substrate-utilization patterns observed in our study support this interpretation. Carbohydrate oxidation remained consistently high across all exercise formats, ranging from 226–228 g·h ⁻ ¹ during the interval and 3000-m trials to approximately 214 g·h ⁻ ¹ during the 30-minute MLaSS run, while fat oxidation was minimal or absent. Statistical analysis confirmed no significant changes in substrate utilization across the 30-minute trial, indicating a stable but markedly elevated glycolytic contribution.

The divergence between our findings and classical lactate-minimum studies may be partly explained by methodological and population-specific differences. Early lactate-minimum research predominantly involved recreational or moderately trained athletes, who typically exhibit lower glycolytic capacity, slower lactate kinetics, and more predictable lactate-nadir responses during incremental exercise [9,25,26]. In contrast, the trained middle-distance runners examined here demonstrated extremely rapid post-sprint lactate appearance, which may distort the early descending limb of the lactate-minimum curve. When lactate clearance temporarily exceeds production, a transient minimum may occur despite the athlete operating above the putative MLSS. This phenomenon has been described in the literature as a false steady state, particularly in athletes with strong anaerobic capabilities [27,28].

Interval-based protocols, such as the 6 × 800-m format used to determine MLaSS, may further contribute to discrepancies. The intermittent nature of this test may create conditions in which lactate removal briefly exceeds lactate production following earlier high-intensity bouts. As a result, the apparent nadir of lactate may represent a recovery-driven minimum rather than a sustainable metabolic steady state. A key physiological reason for the discrepancy may lie in the ability of trained runners to stabilize V̇O₂ despite ongoing lactate accumulation [24,29,30]. Highly trained endurance athletes possess rapid Phase II oxygen kinetics, dense mitochondrial networks, elevated expression of lactate transporters (MCT1/MCT4), and powerful intracellular buffering [11,31,32]. These adaptations may enable them to maintain a steady state in V̇O₂ even when glycolytic flux increases beyond their clearance capacity [33]. Consequently, the runners may *appear* metabolically steady when observing energy utilization data, yet their blood lactate reveals that they are operating well above the true MLSS.

In summary, our study demonstrates that the running velocity derived from the minimum lactate approach did not elicit a stable blood lactate profile during a 30-minute constant-load run in trained middle-distance runners. Although several cardiorespiratory and substrate-utilization variables remained stable, the observed lactate fluctuations indicate that physiological responses at the MLaSS-derived intensity may not reflect a true lactate steady state in this population. Because a classical multi-day MLSS protocol was not performed, these findings should be interpreted as descriptive of MLaSS-related behavior rather than definitive conclusions regarding MLSS.

## 5. Practical application

From a practical perspective, the present findings suggest that in highly trained middle-distance runners, vMLaSS derived from a sprint-induced MLaSS protocol may occur near or above the respiratory compensation point, potentially placing it within the severe intensity domain. Because exercise performed in the upper heavy-severe domain may not achieve a true metabolic steady state, such intensities may not represent a sustainable “threshold” training load. Coaches should therefore exercise caution when prescribing steady-state training based solely on sprint-induced vMLaSS values. Furthermore, the observed dissociation between stable cardiorespiratory variables (V̇O₂ and HR) and fluctuating lactate responses indicates that systemic stability may not necessarily guarantee metabolic steady state. Accordingly, complementary verification strategies (e.g., periodic lactate assessment or performance-based validation) may enhance precision in training prescription.

### 5.1. Study limitation

Our study has several limitations. The most important limitation is that a classical multi-day maximal lactate steady state (MLSS) protocol was not performed. Consequently, the present findings cannot be used to determine whether the running intensity derived from the minimum lactate approach corresponds to MLSS. Instead, the results describe lactate and cardiorespiratory behavior at the workload identified by the MLaSS procedure. Although classical MLSS criteria were used as a reference framework to evaluate lactate stability during the 30-minute constant-load run, these criteria do not constitute direct confirmation of MLSS in the absence of repeated verification trials. A second limitation is that the sample consisted exclusively of trained male runners. Sex-specific differences in aerobic capacity, glycolytic potential, hormonal regulation, and muscle metabolism may influence lactate kinetics and steady-state behavior. Future studies should incorporate both male and female athletes to improve the generalizability of these findings. Additionally, the present protocol employed a sprint-induced hyperlactatemia approach (500-m maximal sprint) combined with a relatively short 8-minute passive recovery. This configuration may influence subsequent lactate kinetics and the position of vMLaSS relative to physiological intensity domains. Therefore, the present findings are specific to this sprint-induced lactate minimum configuration and may not generalize to protocols using different induction distances, recovery durations, or pacing structures.Finally, while continuous cardiopulmonary measurements provided valuable insight into systemic physiological responses, these variables represent indirect indicators of underlying metabolic regulation. Stable oxygen uptake and ventilatory responses do not necessarily reflect intracellular metabolic steady state, particularly in highly trained athletes. Future research should incorporate direct assessments of metabolic function, such as muscle oxygenation, intracellular energetics, or repeated blood-based metabolic measurements, alongside full MLSS verification protocols to more comprehensively characterize steady-state exercise metabolism.

## References

[1] Wasserman K, Whipp BJ, Koyl SN, Beaver WL. Anaerobic threshold and respiratory gas exchange during exercise. J Appl Physiol. 1973;35(2):236–43. doi: 10.1152/jappl.1973.35.2.236 4723033

[2] Wasserman K, Hansen JE, Sue DY, Whipp BJ, Froelicher VF. Principles of exercise testing and interpretation. J Cardiopulm Rehabil Prev. 1987;7(4):189.

[3] Whipp BJ, Ward SA, Wasserman K. Respiratory markers of the anaerobic threshold. Advances in Cardiology. 1987;35:47–64. 10.1159/000413438 3551516

[4] Tegtbur U, Busse MW, Braumann KM. Estimation of an individual equilibrium between lactate production and catabolism during exercise. Med Sci Sports Exerc. 1993;25(5):620–7. doi: 10.1249/00005768-199305000-00015 8492691

[5] Jones AM, Doust JH. The validity of the lactate minimum test for determination of the maximal lactate steady state. Med Sci Sports Exerc. 1998;30(8):1304–13. doi: 10.1097/00005768-199808000-00020 9710874

[6] Simões HG, Denadai BS, Baldissera V, Campbell CSG, Hill DW. Relationships and significance of lactate minimum, critical velocity, heart rate deflection and 3 000 m track-tests for running. J Sports Med Phys Fitness. 2005;45(4):441–51. 16446674

[7] Simões HG, Grubert Campbell CS, Kokubun E, Denadai BS, Baldissera V. Blood glucose responses in humans mirror lactate responses for individual anaerobic threshold and for lactate minimum in track tests. Eur J Appl Physiol Occup Physiol. 1999;80(1):34–40. doi: 10.1007/s004210050555 10367721

[8] Messias LHD, Gobatto CA, Beck WR, Manchado-Gobatto FB. The Lactate Minimum Test: Concept, Methodological Aspects and Insights for Future Investigations in Human and Animal Models. Front Physiol. 2017;8:389. doi: 10.3389/fphys.2017.00389 28642717 PMC5463055

[9] MacIntosh BR, Esau S, Svedahl K. The lactate minimum test for cycling: estimation of the maximal lactate steady state. Can J Appl Physiol. 2002;27(3):232–49. doi: 10.1139/h02-014 12180316

[10] Whipp BJ, Ward SA, Rossiter HB. Pulmonary O2 uptake during exercise: conflating muscular and cardiovascular responses. Med Sci Sports Exerc. 2005;37(9):1574–85. doi: 10.1249/01.mss.0000177476.63356.22 16177611

[11] Beaver WL, Wasserman K, Whipp BJ. A new method for detecting anaerobic threshold by gas exchange. J Appl Physiol (1985). 1986;60(6):2020–7. doi: 10.1152/jappl.1986.60.6.2020 3087938

[12] Sietsema KE, Stringer WW, Sue DY, Ward S. Wasserman & Whipp’s: principles of exercise testing and interpretation: including pathophysiology and clinical applications. Lippincott Williams & Wilkins. 2020.

[13] Bosco C, Luhtanen P, Komi PV. A simple method for measurement of mechanical power in jumping. Eur J Appl Physiol Occup Physiol. 1983;50(2):273–82. doi: 10.1007/BF00422166 6681758

[14] Silva VS, Vieira MFS. International Society for the Advancement of Kinanthropometry (ISAK) Global: International Accreditation Scheme of the Competent Anthropometrist. Revista Brasileira de Cineantropometria & Desempenho Humano. 2020;22:e70517.

[15] Jones AM, Doust JH. A 1% treadmill grade most accurately reflects the energetic cost of outdoor running. J Sports Sci. 1996;14(4):321–7. doi: 10.1080/02640419608727717 8887211

[16] Frayn KN. Calculation of substrate oxidation rates in vivo from gaseous exchange. J Appl Physiol Respir Environ Exerc Physiol. 1983;55(2):628–34. doi: 10.1152/jappl.1983.55.2.628 6618956

[17] Cohen J. The effect size. 1988.

[18] Burgomaster KA, Howarth KR, Phillips SM, Rakobowchuk M, Macdonald MJ, McGee SL, et al. Similar metabolic adaptations during exercise after low volume sprint interval and traditional endurance training in humans. J Physiol. 2008;586(1):151–60. doi: 10.1113/jphysiol.2007.142109 17991697 PMC2375551

[19] Archacki D, Zieliński J, Ciekot-Sołtysiak M, Zarębska EA, Kusy K. Sex Differences in the Energy System Contribution during Sprint Exercise in Speed-Power and Endurance Athletes. J Clin Med. 2024;13(16). 10.3390/jcm13164812 PMC11355823 39200953

[20] Faude O, Kindermann W, Meyer T. Lactate threshold concepts: how valid are they?. Sports Med. 2009;39(6):469–90. doi: 10.2165/00007256-200939060-00003 19453206

[21] Jamnick NA, Pettitt RW, Granata C, Pyne DB, Bishop DJ. An Examination and Critique of Current Methods to Determine Exercise Intensity. Sports Med. 2020;50(10):1729–56. doi: 10.1007/s40279-020-01322-8 32729096

[22] Carter H, Jones AM, Barstow TJ, Burnley M, Williams CA, Doust JH. Oxygen uptake kinetics in treadmill running and cycle ergometry: a comparison. J Appl Physiol (1985). 2000;89(3):899–907. doi: 10.1152/jappl.2000.89.3.899 10956332

[23] Pringle JSM, Doust JH, Carter H, Tolfrey K, Campbell IT, Sakkas GK, et al. Oxygen uptake kinetics during moderate, heavy and severe intensity “submaximal” exercise in humans: the influence of muscle fibre type and capillarisation. Eur J Appl Physiol. 2003;89(3–4):289–300. doi: 10.1007/s00421-003-0799-1 12736837

[24] Burnley M, Jones AM. Oxygen uptake kinetics as a determinant of sports performance. European Journal of Sport Science. 2007;7(2):63–79. doi: 10.1080/17461390701456148

[25] Wahl P, Manunzio C, Vogt F, Strütt S, Volmary P, Bloch W, et al. Accuracy of a Modified Lactate Minimum Test and Reverse Lactate Threshold Test to Determine Maximal Lactate Steady State. J Strength Cond Res. 2017;31(12):3489–96. doi: 10.1519/JSC.0000000000001770 28033123

[26] Dantas De Luca R, Rocha R, Burini RC, Coelho Greco C, Denadai BS. The lactate minimum test protocol provides valid measures of cycle ergometer VO(2)peak. J Sports Med Phys Fitness. 2003;43(3):279–84. 14625507

[27] Zagatto AM, Padulo J, Müller PTG, Miyagi WE, Malta ES, Papoti M. Hyperlactemia induction modes affect the lactate minimum power and physiological responses in cycling. J Strength Cond Res. 2014;28(10):2927–34. doi: 10.1519/JSC.0000000000000490 24736777

[28] Emhoff CAW, Messonnier LA. Concepts of lactate metabolic clearance rate and lactate clamp for metabolic inquiry: a mini-review. Nutrients. 2023;15(14):3213. 37513631 10.3390/nu15143213 PMC10385598

[29] Draper SB, Wood DM. The oxygen uptake response of sprint- vs. endurance-trained runners to severe intensity running. J Sci Med Sport. 2005;8(2):233–43. doi: 10.1016/s1440-2440(05)80014-3 16075783

[30] Poole DC, Jones AM. Oxygen uptake kinetics. Compr Physiol. 2012;2(2):933–96. doi: 10.1002/cphy.c100072 23798293

[31] Jones AM, Poole DC. Oxygen uptake kinetics in sport, exercise and medicine. Routledge. 2013.

[32] Molinari CA, Edwards J, Billat V. Maximal Time Spent at VO2max from Sprint to the Marathon. Int J Environ Res Public Health. 2020;17(24):9250. doi: 10.3390/ijerph17249250 33321958 PMC7763525

[33] Hill DW. Energy system contributions in middle-distance running events. J Sports Sci. 1999;17(6):477–83. doi: 10.1080/026404199365786 10404496

